# The effect of body weight-supported Tai Chi Yunshou on upper limb motor function in stroke survivors based on neurobiomechanical analysis: a four-arm, parallel-group, assessors-blind randomized controlled trial protocol

**DOI:** 10.3389/fneur.2024.1395164

**Published:** 2024-07-09

**Authors:** Liying Zhang, Jiening Wang, Huanxia Zhou, Wangsheng Liao, Naizhen Wang, Xiaoming Yu

**Affiliations:** ^1^Department of Rehabilitation, Seventh People’s Hospital of Shanghai University of Traditional Chinese Medicine, Shanghai, China; ^2^Department of Neurology, Fujian Provincial Governmental Hospital, Fujian, China; ^3^Department of Rehabilitation, Fuzhou Second General Hospital, Fujian, China

**Keywords:** stroke, body weight support, Tai Chi Yunshou, upper limb, motor function, neurobiomechanics

## Abstract

**Introduction:**

A series of functional disorders commonly occur after stroke, of which upper limb dysfunction is the most difficult to recover. The upper limb rehabilitation effect of Tai Chi Yunshou(TCY) in the later stage of stroke has been confirmed by research. Body weight support-Tai Chi Yunshou (BWS-TCY) is based on TCY exercise and robotic exoskeletons offers most flexibility in deweighting and control strategy. This study is aimed to explore the effect of BWS-TCY on upper limb motor function in stroke based on neurobiomechanics.

**Methods and analysis:**

A single-blind randomized controlled trial will be conducted on 36 stroke survivors who will be randomly assigned to three groups: experimental group, control group A and control group B. In addition, 12 healthy elderly people will be recruited into the healthy control group. Those in the experimental group will receive 20 min of CRT and 20 min of BWS-TCY training, while participants in the control group A will receive 20 min of CRT and 20 min of Robot-assisted training. Participants in the control group B will undergo 40 min of Conventional rehabilitation training (CRT) daily. All interventions will take place 5 days a week for 12 weeks, with a 12-week follow-up period. No intervention will be carried out for the healthy control group. Upper limb function will be assessed before and after the intervention using various rating scales (Fugl-Meyer Assessment, Wolf Motor Function Test, etc.), as well as neurobiomechanical analyses (surface electromyography, functional near-infrared brain function analysis system, and Xsens maneuver Capture System). Additionally, 10 healthy elderly individuals will be recruited for neurobiomechanical analysis, and the results will be compared with those of stroke survivors.

**Discussion:**

The results of this study will offer initial evidence on the effectiveness and feasibility of BWS-TCY as an early intervention for stroke rehabilitation. Positive findings from this study could contribute to the development of guidelines for the use of BWS-TCY in the early stages of stroke.

**Ethics and dissemination:**

This study has been approved by the Research Ethics Committees of the seventh People’s Hospital Affiliated to Shanghai University of Traditional Chinese Medicine (Study ID: 2022-7th-HIRB-022). The results of the study will be published in a peer-reviewed journal and presented at scientific conferences.

**Clinical trial registration:**

https://clinicaltrials.gov/, ChiCTR 2200063150.

## Introduction

Stroke is the second most common cause of death and major cause of disability worldwide (1, 2). According to the World Health Organization (WHO), approximately 15 million people suffer from stroke worldwide yearly, more than 5 million die from stroke, and another 5 million suffer from permanent and serious disability (3). In addition, stroke usually leads to serious consequences in survivors, such as neuropsychiatric disorders and impairments in motor, sensory and cognitive abilities (4, 5). Unilateral upper extremity motor dysfunction is among the most common complications (6, 7). Those who suffer from upper-limb dysfunction show limited joint mobility, weakened muscle strength, and coordination disorder. The post-stroke upper-limb dysfunctions can thus limit daily life activities, such as eating, dressing and washing (8–10), increase patient’s dependency and affect the long-term quality of life (11). Therefore, rehabilitation of upper-limb dysfunction is critical to improving their living abilities (12).

By leveraging different rehabilitation technologies, various therapies are applied and used to restore upper-limb functions, such as repetitive transcranial magnetic stimulation (rTMS), transcranial direct current stimulation (tDCS) and so on (13, 14), task-oriented training (15), bilateral training (16), constraint-induced movement therapy (17), virtual reality (18), mirror therapy (19), robotic-assisted therapy (RAT) (20) and Tai Chi exercises (21). Therapies involving high-intensity repetitive tasks, such as the RAT, have the best effect on the recovery of upper-limb functions (22); they provide high-intensity repetitive training, good visual feedback and gravity support (23). However, the RAT compensates the affected upper limbs by gravity and thus hinder the patient’s compliance during practice, which affects the treatment outcomes (24). Therefore, body weight support may not be optimal for long-term rehabilitation of post-stroke upper-limb dysfunctions and for improving the psychological well-being of the survivors. Tai Chi is a traditional aerobic exercise in China and its basic movements are called Tai Chi Yunshou (TCY). It is a low-impact moderate-intensity exercise that focuses on upper-limb motor training and its effectiveness on the stability, endurance, coordination and motor functions of stroke has been confirmed (25–27). TCY is different from other sports interventions, it emphasizes the highly coordinated and complex motor control and hand-eye coordination of the upper limbs (28). Under this kind of exercise, the motor cortex and prefrontal cortex of the brain are activated to a higher degree, which in the long run will lead to the enhancement or remodeling of the functional connections of brain regions (29, 30). However, completing the TCY movement requires better motor functions, such as muscle strength and joint mobility (31, 32). In addition, the completion of TCY exercise generally needs to be in the Brunnstrom stage IV or late of stroke. Therefore, simple and not limited by functional impairment interventional approaches should be developed and involved in current stroke rehabilitation programs, to allow the survivors to persist in the training to obtain ongoing benefits from the therapy.

Based on the above benefits, we will design a set of programs to enable the robot to drive the limited arm to complete the TCY movement. Thus, body weight support-Tai Chi Yunshou (BWS-TCY) may be a proper exercise to improve stroke survivors’ upper limb functions because of its unique motor styles. Firstly, in the early stages of stroke rehabilitation, body weight support is frequently necessary to counteract the impact of gravity on the upper limbs, enabling patients with limited muscle activity to stimulate muscle contraction so that it can actively move as early as possible (24). This creates a beneficial feedback mechanism that promotes brain plasticity and facilitates the restoration of motor function. Secondly, participants need to maintain upper limb stability (movements are smooth and rhythmic) during TCY, making TCY an effective way to stimulate upper limb muscle contractions (33, 34). Thirdly, the survivors strive to control their speed and frequently change the movement angles of various joints during training (25). Performing the TCY could train the coordinative movements and improve the range of motion (35). Furthermore, the survivors need to recall and retrieve these movements while training, which may improve survivors’ cognitive abilities, hand-eye coordination and sense of realism. Finally, robot system could provide good visual feedback and vivid and interesting animation in order to improve participation and motivation (36). Therefore, the TCY holds great potential for facilitating the recovery of upper-limb functions and for improving the mental state of stroke survivors.

Given the potential benefits of BWS-TCY on upper limb motor function in stroke survivors, a pilot experiment was previously conducted. Ninety stroke survivors (Brunnstrom stage ≤ IV) were recruited and evenly divided into three groups. Each group received interventions 5 days a week for 12 weeks. The experimental group performed BWS-TCY and routine rehabilitation training (CRT) for 20 min each daily, control group A performed RAT and CRT for 20 min each daily, and control group B performed CRT for 40 min daily. Evaluation of upper limb motor function was done using Fugl–Meyer motor assessment of upper extremity (FMA-UE) and Wolf Motor Function Test (WMFT), while daily living ability was assessed using Modified Bathel Index (MBI). Preliminary results indicate that BWS-TCY shows promise in improving upper limb motor function and daily living ability, outperforming control group A and control group B (For specific data, please refer to Supplementary material).

In light of the initial experimental findings, further investigation into the mechanism of BWS-TCY in enhancing upper limb motor function in stroke survivors is warranted. Impaired motor function is attributed not only to dysfunction of the central motor control system, but also to muscle synergistic contraction (37). Muscle synergy involves the coordinated activity of agonist and antagonist muscles around the same joint (38, 39). Surface electromyography (sEMG) can detect muscle activity in these muscles and help identify abnormalities in muscle coordination in stroke survivors (40). Functional near-infrared spectroscopy (fNIRS) is a non-invasive imaging technology that relies on optical principles (41). By utilizing the deep tissue penetration of near-infrared light (650 ~ 950 nm), fNIRS can effectively reach 2 ~ 3 cm into the intracranial cerebral cortex (42). Through the correlation between light attenuation and changes in chromophore concentrations in tissue, fNIRS can quantitatively analyze the fluctuations in oxygen-Hb and deoxy-Hb concentrations in brain tissue (43). The functional activities of the brain, driven by the neurovascular coupling mechanism, lead to the activation of the cerebral cortex, resulting in alterations in local blood oxygen metabolic rate and cerebral hemodynamics, ultimately leading to increased local cerebral blood flow (44). The activation area of the brain experiences a much higher increase in oxygen-Hb concentration and a decrease in deoxy-Hb concentration compared to the local oxygen consumption rate (45). As a result, fNIRS can indirectly monitor the functional activity of the cerebral cortex by observing changes in oxygen-Hb and deoxy-Hb concentrations. By correlating brain activation patterns with motor tasks, we can observe the brain’s control strategy for movement, thereby shedding light on the mechanism of BWS-TCY in enhancing upper limb motor function in stroke survivors.

We have introduced a novel intervention utilizing BWS-TCY to investigate the rehabilitative impact and underlying mechanism on individuals who have experienced a stroke. Our aim is to offer valuable insights for healthcare professionals when selecting appropriate clinical rehabilitation training programs for stroke survivors.

## Methods

### Study design

**Figure 1:**
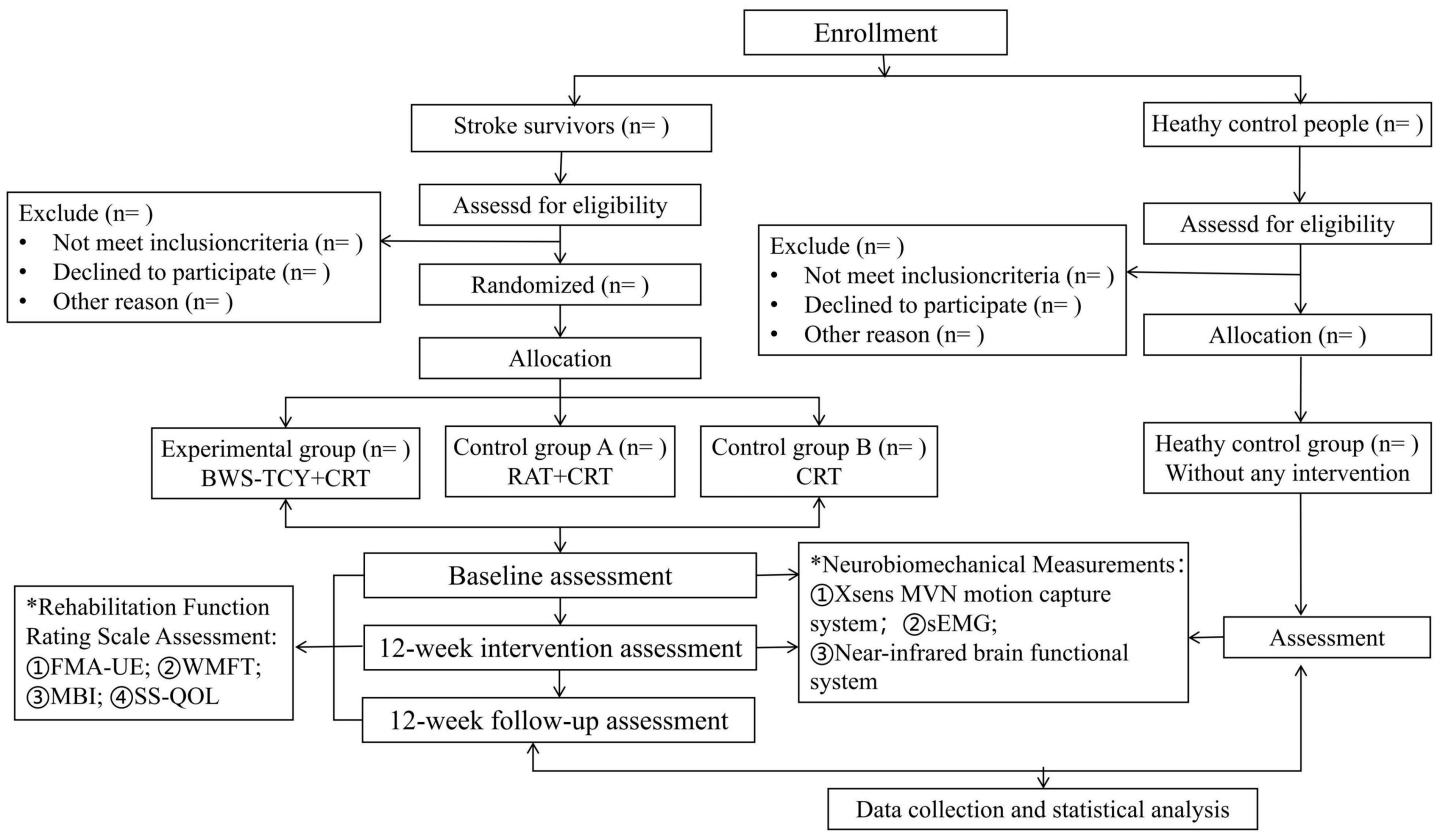
Flowchart of study design.

This study will be a single-center, four-arm, parallel-group, assessors-blind randomized controlled trial. Prior to subject recruitment, all participants will receive information about the study. Those who meet the inclusion criteria and agree to participate will provide informed consent. Following the study flow chart (Figure 1), stroke survivors will be randomly assigned to three groups with equal sample sizes: ① Experimental group (BWS-TCY + CRT group); ② Control group A (RAT + CRT group); and ③ Control group B (CRT group). In addition, a healthy control group (without any intervention) will be set up. The rehabilitation interventions will span 12 weeks. In addition, our study will recruit 12 healthy older adults to take part in research using functional near-infrared brain imaging, surface electromyography, and Xsens motion capture system testing. The findings will be compared with those of stroke survivors both before and after the intervention. Approval for the study protocol has been granted by the institutional review board of Shanghai Seventh People’s Hospital (NO:2022-7th-HIRB-022). The Chinese Clinical Trial Registration Number is ChiCTR 2,200,063,150.

### Study setting

From March 2024 to August 2024, the Neurorehabilitation Department and Neurology Department of Shanghai Seventh People’s Hospital will recruit stroke survivors by reviewing electronic medical records, while the Health Management Department will recruit healthy elderly individuals through physical examination reports.

#### Eligibility criteria for stroke survivors

##### Inclusion criteria

1. Presence of stroke documented by CT or MRI, including ischemic and hemorrhagic stroke;
2. Patients in the sub-acute phase (period 2–12 weeks);
3. Able to sit without upper-limb support;
4. Stable blood pressure (below 160/100 mmHg);
5. Good cognitive ability (Mini-Mental State Exam scores ≥24) (46);
6. No serious visual impairment or visual field defect;
7. 35–80 years old, gender is not limited.

##### Exclusion criteria

1. Other neurological diseases or upper-limb surgical histories;
2. Severe communication deficits;
3. Obvious shoulder pain (pain rating at rest >5) (47);
4. Course of the disease beyond 12 weeks from onset;
5. Stroke survivors who are participating in other clinical studies.

#### Eligibility criteria for healthy volunteers

##### Inclusion criteria

1. Elderly healthy people able to participate and cooperate in the execution of protocol;
2. 60–80 years old, gender is not limited;
3. Elderly people who are in good physical condition and have no underlying diseases that affect exercise.

##### Exclusion criteria

1. Neurological, cardiovascular system and other diseases or upper-limb surgical histories;
2. Obvious shoulder pain (pain rating at rest >5).

### Sample size

G*Power software^1^ will be utilized to determine the sample size for this study, with FMA-UE as the primary outcome measure. Before starting the formal experiment, 30 eligible patients will be divided into groups in a 1:1:1 ratio. The sample size will then be calculated for the primary objective, which is to assess the therapeutic effect after 12 weeks of intervention. Results from the preliminary experiment revealed that after 12 weeks of intervention, the mean scores for the experimental group, control group A, and control group B were 52.45 ± 8.91, 48.93 ± 6.51, and 40.65 ± 10.27, respectively. Based on the G*Power two-factor repeated measures analysis of variance (ANOVA) F test, a total sample size of 30 cases will be deemed necessary for a two-tailed test with 80% power and a significance level of 5% (alpha error). Considering a 1:1:1 allocation ratio and a 20% dropout rate, a final estimation of 36 patients (12 per group) will be determined. Based on the sample size of stroke survivors, we will recruit 12 healthy elderly people.

### Interventions

All healthy elderly people do not participate in any intervention programs. All the stroke survivors will receive rehabilitation interventions based on routine treatment and daily nursing in the hospital. The rehabilitation interventions will be carried out 5 days per week for 12 weeks. Exercise intensity is adjusted according to the data shown on the training device, such as the amount of work done, blood pressure, and heart rate, taking into consideration the individual circumstances of the patient. We will monitor heart rate during the training between groups to determine training intensity. It is typically determined by the heart rate falling within the 70–85% range of (220-age). To improve adherence to training, therapists constantly emphasize the significance and benefits of training to participants and regularly conduct quality assessments. Routine neurological treatment will be permitted. The patient’s safety status will be continuously recorded.

**Figure 2:**
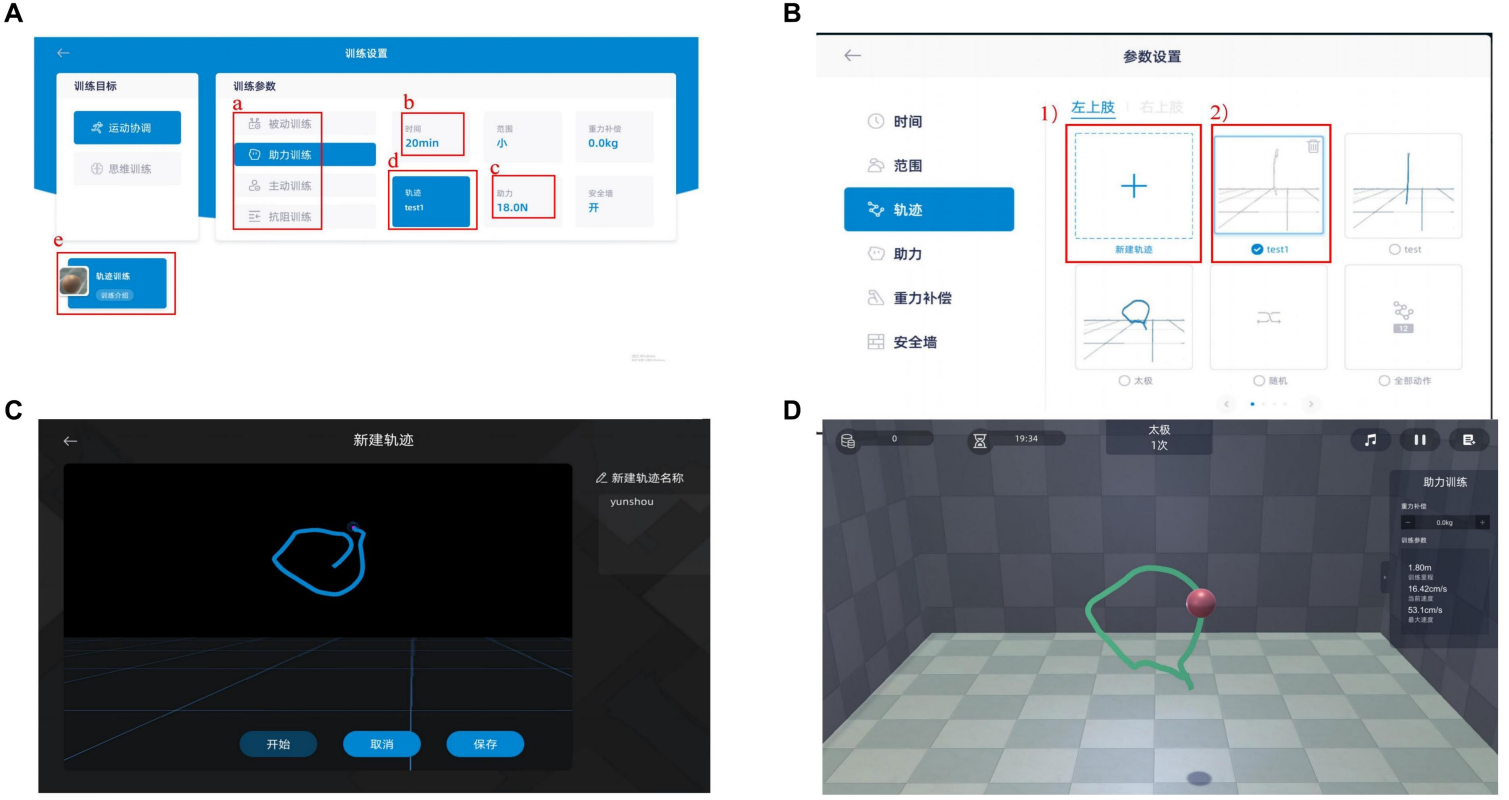
Systerm program of BWS-TCY **(A)** Training parameter; **(B)** Motion path selection; **(C)** Motion trajectory rendering; **(D)** Follow the existing motion path training. **(A)** Selecting training mode; **(B)** Determining training duration; **(C)** Choosing power value; **(D)** Choosing the trajectory of motion; (1) Ploting a new trajectory; (2) Choosing the established trajectory The BWS-TCY system operation processes as follows. Upon selecting a patient from the patient list, the therapist will be directed to the interface depicted in Figure A. This interface includes training parameters that the patient must set before commencing formal training, such as a. Training mode (including passive training, Assistance training, active training, and resistance training), b. Training time, c. Assistance value, and d. Movement trajectory. If the patient is undergoing training for the first time, they are required to sketch a standardized TCY motion trajectory. By clicking on the trajectory option in Figure A, the therapist can access the interface shown in Figure B. Here, the therapist can either create a new motion trajectory for the patient or utilize an existing one. Prior to sketching, the therapist must specify whether the restricted upper limb is on the left or right side, followed by entering the interface displayed in Figure C to begin crafting a new motion trajectory. Upon completion, clicking Save enables direct usage in the future. Finally, by selecting e. Trajectory Training in Figure A, the patient can access the interface depicted in Figure D for TCY training.

**Figure 3:**
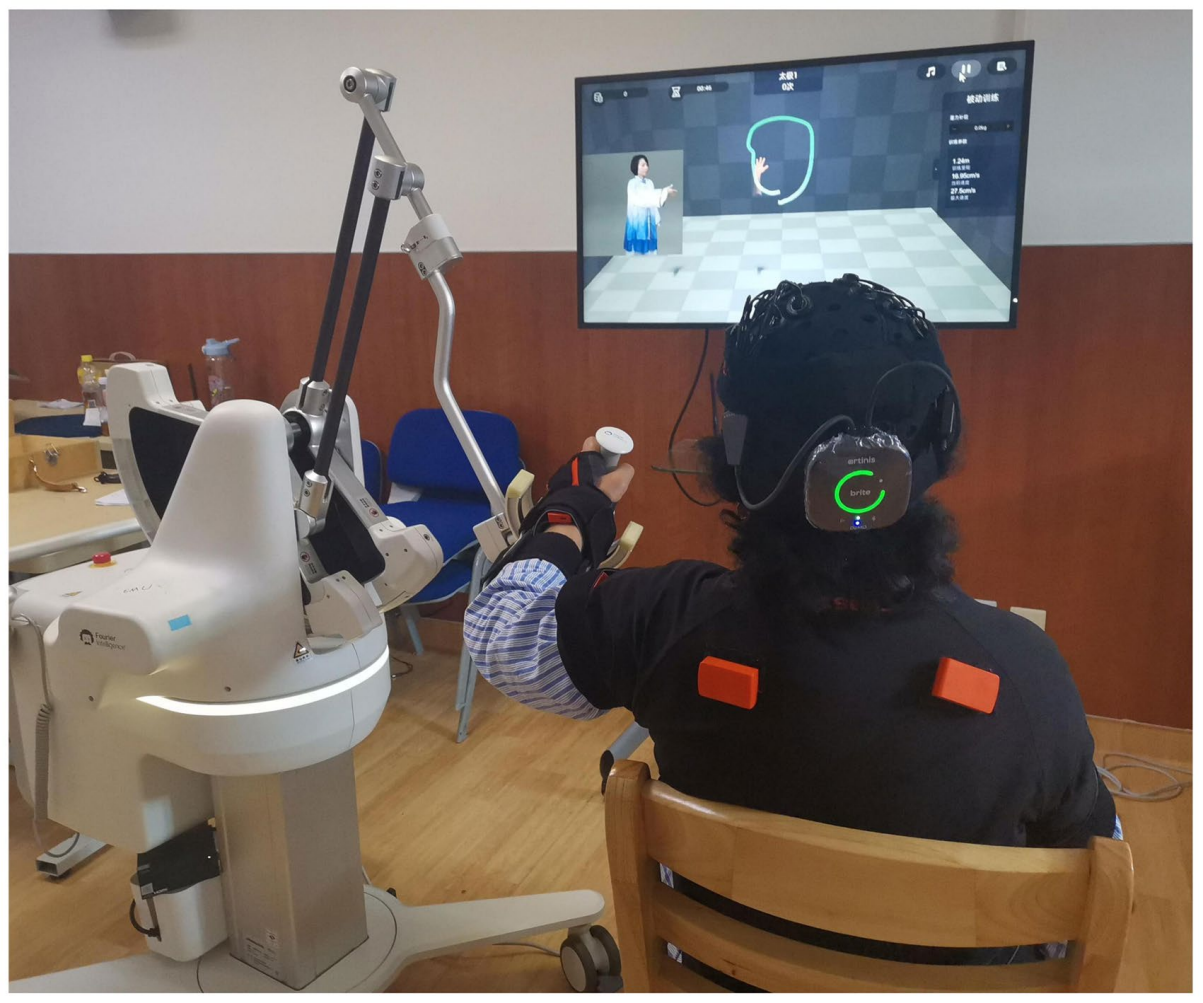
Typical participants practice scenarios of BWS-TCY.

Patients in the BWS-TCY + CRT group will undergo daily sessions consisting of 20 min of CRT treatment and 20 min of BWS-TCY training. The BWS-TCY training will involve watching instructional videos and receiving guidance from therapists trained in TCY movements. A customized TCY movement trajectory program (See Figure 2) will be utilized to assist patients in performing the movements with the help of the exoskeleton rocker of the rehabilitation robot. Patients will be instructed to perform exercises without experiencing upper limb pain. During training, patients will be advised to sit upright, relax their body, maintain proper posture, and focus on the screen displaying the TCY movements. The affected forearm will be secured on the handle in an extended position, and the pre-saved TCY motion trajectory facilitated the completion of the movements using the robot’s mechanical arm. The intervention program will span 12 weeks, progressing from easier to more challenging stages, with varying levels of intensity. Body weight support is provided by the robot’s mechanical arm, which is attached to the forearm of the restricted upper limb. It can offer gravity compensation at various movement angles, adjusting to the movement of the restricted upper limb. This program will include six stages with different weight-bearing percentages for each week: Weeks 1–2: 100%, Weeks 3–4: 80%, Weeks 5–6: 60%, Weeks 7–8: 40%, Weeks 9–10: 20%, Weeks 11–12: 0%. Figure 3 illustrates typical participants practice scenarios of BWS-TCY.

**Figure 4:**
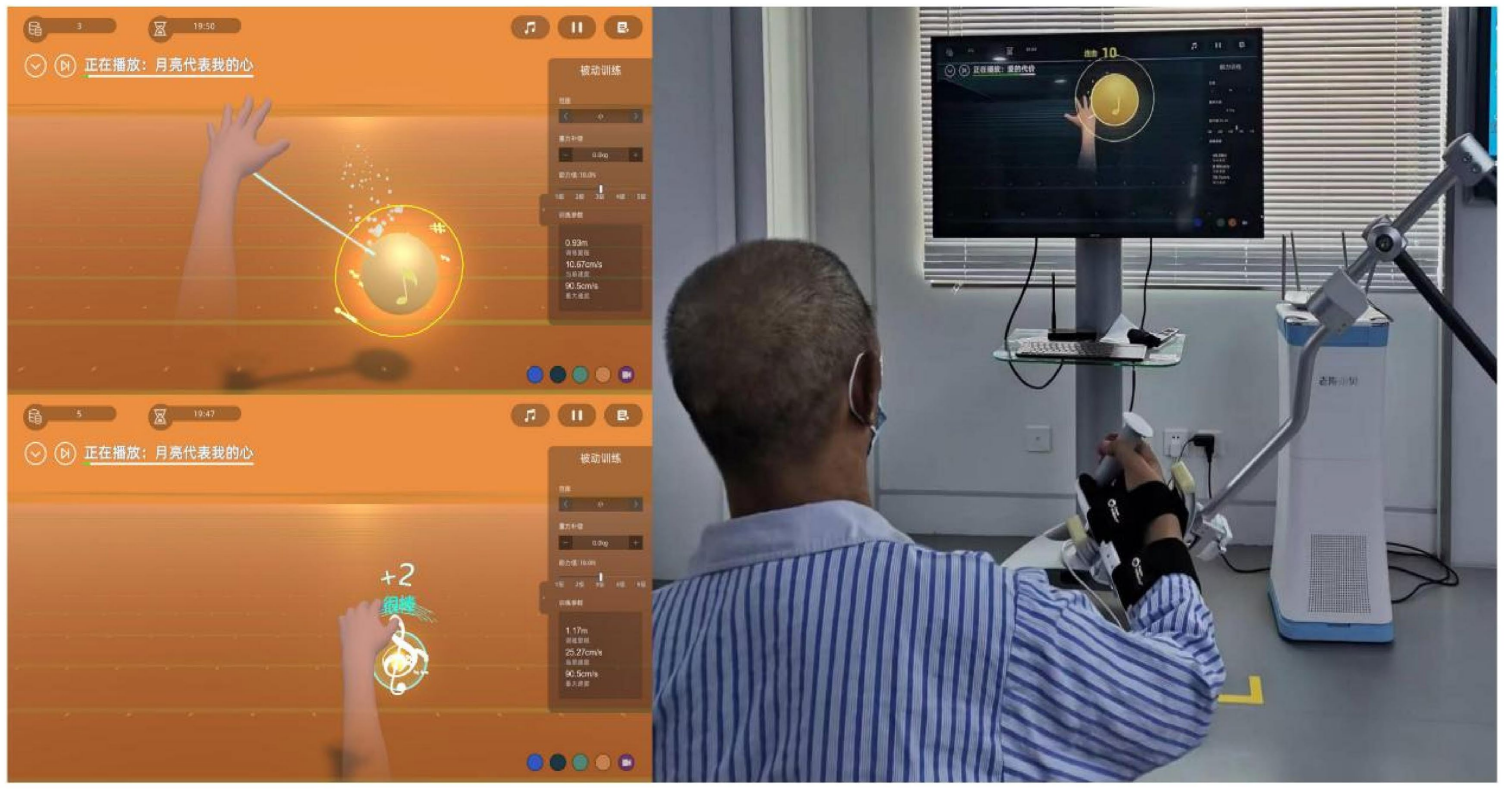
Robot-assisted training scenario: note-grabbing game.

Patients in the RAT+CRT group will receive 20 min of CRT treatment and 20 min of RAT training daily. The RAT training will be conducted using the Fourier ArmMotus EMU device, a robot equipped with weight support and offering passive, assistive, and active modes based on the patient’s functional status. The focus of RAT is primarily on the dysfunctional side, involving training the upper limb of the hemiplegic side with the assistance of the rehabilitation robot’s exoskeleton. This training will include capturing musical notes displayed on the screen in various random and unpredictable directions (refer to Figure 4 for the training scenario).

Participants in CRT group will receive 40 min of CRT daily, with sessions lasting 20 min each in the morning and afternoon. The treatment primarily will targete the hemiplegic side through physical therapy, occupational therapy, and rehabilitation care. Specific training activities will include: ① Proper limb positioning; ② Bed mobility training; ③ Passive range of motion exercises for upper and lower limbs; ④ Bobath handshake and arm raising exercises; ⑤ Rehabilitation training for finger function and fine motor skills; ⑥ Balance exercises; and ⑦ Activities to enhance daily life abilities.

### Assignment of interventions

Subject randomization will be performed by an external professional statistician. Every participant will get an envelope containing a random allocation sequence number to determine which group, after baseline testing. The randomization sequence will be obtained by an independent professional statistician using Excel (Microsoft, United States).

Randomization will be placed in a sealed opaque envelope by an independent researcher who is blinded to the trial. The identity of each participant will be represented by a sequence number. The letters ‘E’, ‘A’, and ‘B’ will represent assigned groups. ‘E’ represents the experimental group, ‘A’ represents the control group A, and ‘B’ represents the control group B. The Investigator will be responsible for recruiting and assigning participants to the intervention.

Specifically, the statistician, outcome assessors and data analyzers will be blinded to study recruitment, intervention and evaluation. Patients, researchers and therapists will not be blinded, as this is not possible due to major differences between groups. The trial will be specified to be open-label, so there will be no blinding of patients.

### Data collection and management

Two assessors will be trained extensively on data collection using electronic case report forms (CRFs). All outcome measures will be assessed before and after the intervention, as well as during follow-up. Participants can withdraw at any time and will be asked for consent regarding the use of their data if they choose to do so. They will also be queried on whether they permit the research team to share relevant data. Data will be recorded on the CRF by the evaluator, with the complete test report attached. The CRF will be entered monthly and managed by an independent organization. Patient identifiable information will be stored separately from clinical information and study data by one of the authors responsible for patient randomization. To safeguard patient confidentiality, only study leaders, authors, and the ethics committee will have access to patients’ personal information and medical records. Upon completion of the study, all data will be stored on a password-protected hard drive.

### Outcome assessment

Outcome measures of upper limb motor function scale:

1. The FMA-UE is the main index used in this study to measure upper limb motor dysfunction after stroke. It is a cost-effective clinical examination method that is widely used in stroke patients due to its reasonable design, simplicity, and ease of use (48). The FMA-UE assesses reflex activities, shoulder, elbow, and wrist joint movement, as well as coordination. It consists of 8 aspects and 33 items, with each item scored on a scale of 0 to 2 points. The total score ranges from 0 to 66 points.
2. The WMFT is utilized to assess the motor function of stroke patients’ upper limbs (49). Unlike the FMA, which primarily assesses stroke patients’ coordination function, the WMFT can measure the impact of injury and training on disability (50). Moreover, it can also indicate the effects of different functional task training on patients. The test comprises 15 items, with the initial 6 items concentrating on basic joint movements and the remaining 9 items involving more complex functional movements. Each movement is timed and rated on a six-point scale from 0 to 5 points based on the quality of the movement.

Outcome measures of activities of daily living and quality of life:

1. The MBI is a widely utilized tool for assessing the daily living capabilities of stroke patients (51). Comprising 10 tasks, this index assigns scores based on the time and level of assistance needed by the patient to perform each task. A total score of 100 points is possible, with lower scores indicating a higher reliance on nursing care. A score of 60 points suggests that the individual is largely capable of self-care.
2. The Stroke- specific Quality of Life (SS-QOL) is a patient-reported tool used to evaluate the health-related quality of life of stroke patients (52). It consists of 49 items across 12 domains, with each domain being scored individually. Each item within a domain can receive a maximum of 5 points, and the total score is then calculated. A higher score indicates better functioning.

Outcome measures of neurobiomechanics:

1. The Xsens MVN Animate motion capture system (Xsens Technologies BV, Enschede, Netherlands) (53) and sEMG equipment (54) will be used to simultaneously collect kinematic and dynamic parameters in the target action from the experimental group, control group A, control group B, and healthy older adults. The motion capture system operates at a sampling frequency of 100 Hz. Following a brief warm-up, participants will be engaged into experimentally standardized dressing activities, including clothes, shoes, and socks. Marker balls were sequentially placed on anatomical landmarks of the body based on guidelines from the Xsens motion capture system manual. Notably, tracking Markers were attached to the humerus and wrist bones. Electrode pads for the surface electromyography device were applied to specific muscles such as the pectoralis major, biceps brachii, triceps brachii, anterior deltoid, middle deltoid, pronator teres, pronator quadratus, and radial wrist extensor. The data will be sampled at 1500 Hz after online bandpass filtering between 5 and 450 Hz. Prior to the formal testing, subjects will undergo 5 pre-tests of the TCY action to familiarize themselves with the experimental setup and objectives. Three successful data sets will be collected during the formal experiment for subsequent processing and analysis. The original motion capture data will be refined and filtered using Xsens MVN Analyze software, then imported into Visual 3D (C-motion, United States) for further computation and analysis. Parameters such as integrated electromyography (iEMG), average electromyography (aEMG), and root mean square (RMS) will be calculated based on the sEMG data. The sEMG signal will undergo high-pass filtering with a cutoff frequency of 20 Hz and will be then corrected using the Hilbert transform. Subsequently, the rectified sEMG signal will be low-pass filtered with a cutoff frequency of 10 Hz to isolate the sEMG envelope. Nonnegative matrix factorization will be employed utilizing the sEMG envelope to extract muscle effects. This process will decompose the sEMG envelope into two non-negative matrices, with one representing synergy and the other indicating the corresponding activation pattern. The number of synergistic muscle modules will be determined using Variance Explained (VAF). For visualization of the functional muscle network, a threshold of 30% of the maximum edge weight in a single adjacency matrix is selected. The network topology will be then depicted using a consistent threshold across all groups, with edges classified based on their strength as low (0–33%), medium (34–66%), or high (67–100%).
2. The LABNNRIS fNIRS system will be used by participants in the experimental group, control group A, control group B, and healthy control group to analyze the levels of oxyhemoglobin, deoxygenated hemoglobin, and total hemoglobin in the cerebral cortex to assess brain activity (55). Specifically, they focused on observing changes in the premotor cortex (PMC) and supplementary motor cortex (SMC). The researchers will select block module settings, defined the task and rest states (usually 30s each), and calculated the oxyHb levels during the task compared to the rest. The activation of each cortical area will be determined as the average of the corresponding channel activation. The hemoglobin signal will be sampled at a rate of 0.13 s (56), with a data sampling frequency of 10 Hz and a detector-to-light source distance of 30 mm to ensure propagation to the gray matter below the optode (57). Each fNIRS channel will be positioned at the midpoint of the corresponding source-detector pair, with a total of 24 channels constructed from 10 light sources and 16 detectors. These channels will be symmetrically distributed across the participant’s left and right hemispheres (12 channels per side) following the 10/10 International Electrode Placement System. The channels are located in the left and right prefrontal cortex (LPFC/RPFC), the left and right motor cortex (LMC/RMC), and the left and right occipital lobes (LOL/ROL) (56). These regions are associated with motor function and are critical for cognitive processing and motor control (58, 59). The instrument’s calibration function and corresponding templates will be utilized to accurately determine the channels that correspond to the 10/10 electrode positions based on participants’ different head sizes. The data will be analyzed with reference to previous literature (56, 57, 60). Fluctuations in ΔHbO2 and ΔHHb concentrations will be calculated based on changes in detected light intensity using the modified Beer–Lambert law (assuming constant scattering). Following the acquisition of ΔHbO2 and ΔHHb signals, data preprocessing will be performed. A moving average method will be employed to remove obvious outliers in the signal, using a 3-s time window for the filter. Subsequently, processing techniques involving moving standard deviation and cubic spline interpolation will be utilized to eliminate motion artifacts (61). Artifacts will be identified by detecting sliding window standard deviations exceeding a specific threshold and then removed through cubic spline interpolation. A sixth-order Butterworth bandpass filter ranging from 0.021 to 2 Hz will be applied to enhance the signal-to-noise ratio of the filtered signal. For comparing changes in motor performance scores among groups, a one-way ANOVA followed by *post hoc* analysis with Bonferroni correction will be conducted. To explore the relationship between fNIRS data and neurophysiology as well as clinical behavior, Spearman correlation will be used to analyze the values of ΔHbO and RMT. Additionally, Spearman correlation analysis will be employed to investigate the association between the distance of fNIRS-HS from the mean MEP-HS and motor improvement, correlating the distance with changes in motor performance scores within the fNIRS group.

### Participant timeline

The study protocol, including enrolment, intervention and assessment, is shown in Table 1 (recommended for interventional trials (SPIRIT) 2013) (62).

**Table 1:** Example template of recommended content for the schedule of enrollment, interventions, and assessments.

	Study period					
	Enrolment	Allocation	Format intervention			Follow-up
Timepoint**	T_0_	0	T_4_	T_8_	T_12_	T_24_
Enrolment:						
Eligibility screen	**×**					
Informed consent	**×**					
Allocation		×				
Interventions:						
*[CRT]*						
		
*[RAT + CRT]*						
		
*[BWS-TCY + CRT]*						
			
Assessments:						
*Characteristics*	**×**					
*FMA-UE*	**×**		**×**	**×**	**×**	**×**
*WMFT*	**×**		**×**	**×**	**×**	**×**
*Surface* *electromyography*	**×**				**×**	
*Brain Connectomics*	**×**				**×**	
*Xsens motion* *capture system*	**×**				**×**	
*MBI*	**×**		**×**	**×**	**×**	**×**
*SS-QOL*	**×**		**×**	**×**	**×**	**×**

### Statistical method

Statistical analyses will be carried out using SPSS software (IBM Corp., IBM SPSS Statistics, V24, Armonk, NY, United States). Two-way analysis of variance (ANOVA) will be utilized to investigate the interaction and main effects of the intervention method and assessment time. The impact of the intervention will be assessed by comparing changes in upper extremity functionality among groups using analysis of covariance of change scores, with baseline as a covariate and adjustments made for potential confounders. If a significant difference is detected, *post hoc* analysis will be conducted using Tukey’s test. Demographic characteristics and baseline values will be presented using descriptive statistics for each group. A significance level of *p* < 0.05 will be set for all statistical tests, with corrections made for multiple comparisons when necessary. Additionally, a sensitivity analysis in the form of an intention-to-treat (ITT) analysis, analyzing participants in the treatment groups as originally allocated, using observed data only, may be performed.

We will rigorously adhere to regulatory documentation and reporting processes to address any instances of non-compliance and deviations. The ITT analysis will be conducted using the last observation carried forward (LOCF) method to handle missing data. Our focus will be on implementing recruitment and retention strategies to minimize missing data. If data are missing at random, multiple imputation will be utilized to address sporadic absences of random outcomes. For non-ignorable missing data, sensitivity analyses will be conducted using pattern mixture or selection models to assess the robustness of our conclusions under various plausible scenarios.

### Oversight and monitoring

This is a single-center study conducted and coordinated at Shanghai Seventh People’s Hospital. Day-to-day support for the trial will be provided by the following:

1. Principal Investigator: Oversees trial and medical responsibilities for patients.
2. Study Coordinator: Trial registration and coordination of study visits.
3. Research therapists: Identify potential recruits, obtain informed consent and intervene per protocol.

The research team will meet every 2 weeks. There will be no trial steering committee, stakeholder or public engagement group. A data monitoring committee will not be required because of the expected low safety risk for the participants.

Throughout the study, all adverse events will be recorded in the CRF and the occurrence of adverse reactions will be fully analysed and assessed, along with symptomatic treatment and active management of events. Serious adverse events occurring during the study will be reported to the ethics committee within 24 h.

The project management team will report the research progress in the form of bi-weekly research meetings. The Trial Ethics Committee will oversee the trial procedures and recommend changes to the necessary protocol for the study. In this study, the process will be reviewed by means of on-site monitoring.

### Patient and public involvement

The original research ideas were conceived by the authors and adjusted based on input and feedback from stroke patients and rehabilitation therapists to ensure the safety and applicability of the intervention. Before the formal experiment, 30 stroke patients will be invited for BWS-TCY training. The results of this study will be used to identify the strengths and limitations of novel interventions and advocate for improvements in their design and application.

### Ethics and dissemination

All study procedures are in accordance with the Declaration of Helsinki in its current version.^2^ Patients, relatives and their representatives will be given the opportunity to discuss the study protocol and mention concerns not addressed in a draft proposed at the time. Participants will be informed of the study protocol, possible risks and other related matters before entering the study. They will sign the informed consent before randomization. The present study protocol has been approved by the Medical Ethics Committee of Shanghai Seventh People’s Hospital (NO: 2022-7th-HIRB-022). After an initial screening of patients according to inclusion and exclusion criteria, eligible volunteers who agree to participate will sign written informed consent before the intervention. The principal investigator will be responsible for the procedure of collecting the informed consent. On the consent form, the participants will be told of their right to withdraw at any time. Participants will also be asked whether they allow the researchers to share their relevant data. The test will not involve the collection of biological samples for storage.

The results of the study will be published in peer-reviewed scientific journals and presented at conferences and workshops within 12 months after study completion. Individuals who meet the authorship criteria will be listed as authors of the publication, as directed by the International Committee of Medical Journal Editors. BWS-TCY exercises and corresponding equipment (programs, movement trajectories and others) will be optimized and promoted to the majority of physiotherapists to achieve the clinical transition.

## Discussion and conclusion

To ensure that BWS-TCY treatment of upper extremity motor dysfunction is available and safe, all clinical procedures will be performed in the hospital, and treatment will be performed under professional guidance. The American Stroke Association (63) guidelines point out that the earlier the rehabilitation intervention, the better the effect. Therefore, the results of this study will provide preliminary evidence for the effectiveness and feasibility of BWS-TCY as an intervention for early stroke rehabilitation. Studies have shown that stroke patients need to receive interventional rehabilitation as soon as possible after their vital signs have been stabilized (64). If the findings are positive, this study will help to establish further guidelines for the application of BWS-TCY in the early stages of stroke. Results would confirm that BWS-TCY can include stroke patients in the early and flaccid stages, which is more reasonable and closer to clinical practice.

## Ethics statement

The studies involving humans were approved by Ethics Committee of Shanghai Seventh People’s Hospital. The studies were conducted in accordance with the local legislation and institutional requirements. The participants provided their written informed consent to participate in this study. Written informed consent was obtained from the individual(s) for the publication of any potentially identifiable images or data included in this article.

## Author contributions

LZ: Data curation, Formal analysis, Methodology, Software, Writing – original draft, Writing – review & editing. JW: Conceptualization, Data curation, Formal analysis, Funding acquisition, Investigation, Resources, Supervision, Writing – original draft. HZ: Funding acquisition, Methodology, Project administration, Supervision, Visualization, Writing – review & editing. WL: Project administration, Supervision, Validation, Visualization, Writing – review & editing. NW: Conceptualization, Data curation, Funding acquisition, Investigation, Methodology, Project administration, Resources, Writing – review & editing. XY: Conceptualization, Data curation, Formal analysis, Funding acquisition, Methodology, Project administration, Resources, Supervision, Writing – review & editing.

## Footnotes

^1^v3.1.9.2, University Dusseldorf, Germany; available for download from http://www.psychologie.hhu.de

^2^for details, see www.wma.net
