# Supplementary material for: The effect of body weight-supported Tai Chi Yunshou on upper limb motor function in stroke survivors based on neurobiomechanical analysis: a four-arm, parallel-group, assessors-blind randomized controlled trial protocol
Source: Front Neurol. 2024 Jul 9;15:1395164. doi: 10.3389/fneur.2024.1395164 (PMC11263172; doi:10.3389/fneur.2024.1395164)
Supplement: Supplementary file 2 [file Table_1.DOCX]

**Supplementary materials: Pre-experimental statistical analysis results**

| **Table 1** Repeated measurement of Mauchly spherical test for upper limb motor function | | | | | |
| --- | --- | --- | --- | --- | --- |
| Outcome measures | Mauchly W value | Approximate chi-square value | Significance | Degrees of freedom | Greenhouse-Greisser correction |
| FMA-UE | 0.160 | 157.208 | <0.001 | 5 | 0.496 |
| WMFT | 0.236 | 123.722 | <0.001 | 5 | 0.537 |

| **Table 2** Repeated measures analysis of variance for upper limb motor function | | | | | | | |
| --- | --- | --- | --- | --- | --- | --- | --- |
| Outcome measures | Scourses | Sum of Squares | Degrees of freedom | Mean Square | *F* value | Significance | Eta Squared |
| FMA-UE | Time | 19020.42 | 1.487 | 7499.84 | 285.87 | <0.001 | 0.77 |
|  | Group | 1279.92 | 2.957 | 357.57 | 9.62 | <0.001 | 0.18 |
|  | Group×Time | 1955.29 | 2 | 1313.66 | 3.67 | 0.030 | 0.08 |
| WMFT | Time | 18916.43 | 1.612 | 11735.78 | 392.52 | <0.001 | 0.82 |
|  | Group | 839.88 | 3.224 | 260.53 | 8.71 | <0.001 | 0.17 |
|  | Group×Time | 1878.65 | 2 | 939.33 | 4.25 | 0.017 | 0.09 |

**Table 3** Ratings of upper limb motor function in three groups at different time points

| Outcome measures | Groups | Time point | Mean | Standard deviation | Confidence Interval (%) | |
| --- | --- | --- | --- | --- | --- | --- |
|  |  |  |  |  | Upper | Lower |
| FMA-UE | CRT+BWS-TCY | before intervention | 25.28 | 9.27 | 21.75 | 28.80 |
|  |  | 4 weeks after intervention | 35.62 | 8.70 | 32.31 | 38.93 |
|  |  | 8 weeks after intervention | 42.48 | 8.70 | 39.17 | 45.79 |
|  |  | 12 weeks after intervention | 52.45 | 8.91 | 49.06 | 55.84 |
|  | CRT | before intervention | 25.10 | 9.56 | 32.31 | 38.93 |
|  |  | 4 weeks after intervention | 32.16 | 8.69 | 28.97 | 35.35 |
|  |  | 8 weeks after intervention | 35.26 | 8.82 | 32.02 | 38.49 |
|  |  | 12 weeks after intervention | 40.65 | 10.27 | 36.88 | 44.41 |
|  | CRT+RAT | before intervention | 27.73 | 10.76 | 23.71 | 31.75 |
|  |  | 4 weeks after intervention | 34.83 | 8.34 | 31.72 | 37.95 |
|  |  | 8 weeks after intervention | 39.00 | 8.43 | 35.85 | 42.15 |
|  |  | 12 weeks after intervention | 48.93 | 6.51 | 46.50 | 51.36 |
| WMFT | CRT+BWS-TCY | before intervention | 29.17 | 8.68 | 25.93 | 32.41 |
|  |  | 4 weeks after intervention | 34.73 | 8.56 | 28.59 | 33.61 |
|  |  | 8 weeks after intervention | 43.20 | 9.49 | 32.36 | 37.64 |
|  |  | 12 weeks after intervention | 49.10 | 8.58 | 45.89 | 52.31 |
|  | CRT | before intervention | 29.13 | 6.99 | 25.93 | 32.41 |
|  |  | 4 weeks after intervention | 31.10 | 6.73 | 28.59 | 33.61 |
|  |  | 8 weeks after intervention | 35.00 | 7.07 | 32.36 | 37.64 |
|  |  | 12 weeks after intervention | 38.43 | 7.93 | 35.47 | 41.39 |
|  | CRT+RAT | before intervention | 27.97 | 7.52 | 25.16 | 30.78 |
|  |  | 4 weeks after intervention | 33.17 | 7.19 | 30.48 | 35.85 |
|  |  | 8 weeks after intervention | 43.00 | 6.06 | 40.74 | 45.26 |
|  |  | 12 weeks after intervention | 48.97 | 5.57 | 46.89 | 51.05 |

**Table 4** Differences in upper limb motor function between the three groups after the intervention and before the intervention (Mean±SD, score)

| Outcome measures | Groups | Difference between 4 weeks after intervention and before intervention | Difference between 8 weeks after intervention and before intervention | Difference between 12 weeks after intervention and before intervention |
| --- | --- | --- | --- | --- |
| FMA-UE | CRT+BWS-TCY | 10.34±8.84 | 17.21±9.17 | 27.17±9.64 |
|  | CRT | 7.06±8.33 | 10.16±8.71 | 15.55±9.04 |
|  | CRT+RAT | 8.13±8.3 | 11.27±7.29 | 17.30±9.26 |
| WMFT | CRT+BWS-TCY | 8.59±7.41 | 15.69±7.87 | 24.45±6.89 |
|  | CRT | 6.29±7.99 | 9.32±8.42 | 14.81±8.73 |
|  | CRT+RAT | 7.03±7.00 | 12.37±5.28 | 20.30±6.57 |

**Table 5** Comparison of upper limb motor function scores among the three groups after 12 weeks of intervention

| Outcome measures | Comparison between groups | t value | *P* value (two-tailed) | Mean Difference | Standard error value |
| --- | --- | --- | --- | --- | --- |
| FMA-UE | CRT+BWS-TCY *VS* CRT | 4.740 | <0.001 | 11.80 | 2.49 |
|  | CRT+BWS-TCY *VS* CRT+RAT | 1.802 | 0.077 | 4.39 | 2.44 |
|  | CRT+RAT *VS* CRT | 3.242 | 0.002 | 7.41 | 2.89 |
| WMFT | CRT+BWS-TCY VS CRT | 4.720 | <0.001 | 9.64 | 2.02 |
|  | CRT+BWS-TCY VS CRT+RAT | 2.365 | 0.021 | 4.15 | 1.75 |
|  | CRT+RAT VS CRT | 2.770 | 0.007 | 5.49 | 1.97 |

**Table 6** Repeated measurement of Mauchly sphericity hypothesis test for MBI

| Mauchly W value | Approximate chi-square value | Significance | Degrees of freedom | Greenhouse-Greisser correction |
| --- | --- | --- | --- | --- |
| 0.540 | 52.786 | <0.001 | 5 | 0.744 |

**Table 7** Repeated measures analysis of variance of MBI in three groups

| Scourses | Sum of Squares | Degrees of freedom | Mean Square | F value | Significance | Eta Squared |
| --- | --- | --- | --- | --- | --- | --- |
| Time | 28081.49 | 2.232 | 12578.59 | 533.25 | <0.001 | 0.86 |
| Group | 1416.98 | 4.465 | 317.35 | 13.45 | <0.001 | 0.24 |
| Group×Time | 1682.47 | 2 | 841.23 | 1.812 | 0.169 | 0.04 |

**Table 8** MBI scores of three groups at different time points

| Groups | Time point | Mean | Standard deviation | Confidence Interval (%) | |
| --- | --- | --- | --- | --- | --- |
|  |  |  |  | Upper | Lower |
| CRT+BWS-TCY | before intervention | 46.38 | 14.01 | 41.05 | 51.71 |
|  | 4 weeks after intervention | 55.86 | 12.61 | 51.06 | 60.66 |
|  | 8 weeks after intervention | 66.21 | 13.54 | 61.06 | 71.36 |
|  | 12 weeks after intervention | 76.55 | 12.54 | 71.78 | 81.32 |
| CRT | before intervention | 47.74 | 9.99 | 44.08 | 51.01 |
|  | 4 weeks after intervention | 52.42 | 9.56 | 48.91 | 55.93 |
|  | 8 weeks after intervention | 59.81 | 9.41 | 56.36 | 63.26 |
|  | 12 weeks after intervention | 64.52 | 10.83 | 60.54 | 68.49 |
| CRT+RAT | before intervention | 48.33 | 10.61 | 44.37 | 52.30 |
|  | 4 weeks after intervention | 55.33 | 10.98 | 51.23 | 59.43 |
|  | 8 weeks after intervention | 63.67 | 11.29 | 59.45 | 67.88 |
|  | 12 weeks after intervention | 71.67 | 10.37 | 67.80 | 75.54 |

**Table 9** Difference between three groups after MBI intervention and before intervention (Mean±SD, score)

| Groups | Difference between 4 weeks after intervention and before intervention | Difference between 8 weeks after intervention and before intervention | Difference between 12 weeks after intervention and before intervention |
| --- | --- | --- | --- |
| CRT+BWS-TCY | 8.59±7.41 | 9.48±3.86 | 30.17±6.78 |
| CRT | 6.29±7.99 | 4.68±4.82 | 16.77±6.78 |
| CRT+RAT | 7.03±5.35 | 7.00±5.81 | 23.33±7.69 |

**Table 10** Comparison of MBI between three groups after 12 weeks of intervention

| Comparison between groups | t value | *P* value (two-tailed) | Mean Difference | Standard error value |
| --- | --- | --- | --- | --- |
| CRT+BWS-TCY *VS* CRT | 6.98 | <0.001 | 13.39 | 1.92 |
| CRT+BWS-TCY *VS* CRT+RAT | 3.32 | 0.002 | 6.84 | 2.05 |
| CRT+RAT *VS* CRT | 3.54 | 0.001 | 6.56 | 1.86 |
